# Integrating Tumor Budding and the Invasive-Front Microenvironment in Colorectal Carcinoma: An Exploratory Histopathological Score-Based Association Study

**DOI:** 10.3390/ijms27114971

**Published:** 2026-05-30

**Authors:** Valeria Zuccalà, Vincenzo Fiorentino, Teresa Maria Martorana, Gabriele Ricciardi, Pietro Tralongo, Giuliana Ciappina, Vittorio Abbonante, Michele Ammendola, Massimiliano Berretta, Guido Fadda, Maurizio Martini, Antonio Ieni

**Affiliations:** 1 Anatomic Pathology Unit, Department of Human Pathology in Adult and Developmental Age “Gaetano Barresi”, University of Messina, 98125 Messina, Italy; 2 PhD Program in Translational Molecular Medicine and Surgery, Department of Biomedical Sciences, Dental Sciences and Morpho-Functional Imaging, University of Messina, 98125 Messina, Italy; gabriele.ricciardi@studenti.unime.it (G.R.);; 3 Istituto Clinico Polispecialistico C.O.T. Cure Ortopediche Traumatologiche s.p.a., 98124 Messina, Italy; 4 Section of Experimental Medicine, Department of Medical Sciences, University of Ferrara, 44121 Ferrara, Italy; giuliana.ciappina@unife.it; 5 Department of Health Sciences, Magna Graecia University of Catanzaro, 88100 Catanzaro, Italy; 6 Department of Experimental and Clinical Medicine, Magna Graecia University of Catanzaro, 88100 Catanzaro, Italy; michele.ammendola@unicz.it; 7 Medical Oncology Unit, Department of Clinical and Experimental Medicine, University of Messina, 98125 Messina, Italy

**Keywords:** colorectal carcinoma, invasive tumor front, tumor budding, podoplanin, tumor microenvironment, tumor-associated macrophages, mast cells, angiogenesis, microvessel density, histopathological score

## Abstract

The invasive tumor front (ITF) of colorectal carcinoma (CRC) is a biologically active interface where epithelial dissociation, stromal activation, immune remodeling, and angiogenesis converge. This retrospective histopathological association study examined whether tumor budding combined with selected ITF microenvironmental markers could better characterize aggressive clinicopathological features of non-metastatic CRC. Fifty-two surgically resected primary CRCs were analyzed after exclusion of patients with distant metastatic disease at diagnosis or surgery. Tumor budding was scored according to ITBCC 2016 criteria. Immunohistochemistry evaluated stromal podoplanin (D2-40) expression, CD34-based microvessel density, the CD163/CD68 ratio, and CD117-positive mast cell density. An unweighted integrated ITF microenvironment score (0–4) was generated by summing high values for these four parameters. High histological grade, lymphovascular invasion, high tumor budding, and node-positive disease were significantly associated with multiple microenvironment-related alterations. The integrated ITF score was significantly higher in high-grade, lymphovascular invasion-positive, node-positive, and high-budding CRCs, suggesting cumulative stromal, immune, and vascular remodeling at the invasive front. These findings support the ITF as an integrated histopathological compartment in CRC progression. However, the score remains hypothesis-generating and requires validation in larger, independent, molecularly and outcome-annotated cohorts before prognostic or clinical use.

## 1. Introduction

Colorectal carcinoma (CRC) is increasingly recognized as a complex and dynamic disease arising from reciprocal interactions between neoplastic epithelial cells and the surrounding microenvironment, rather than being driven solely by tumor cell-intrinsic alterations [1,2,3]. Within this framework, the tumor microenvironment (TME) represents a highly organized system composed of stromal elements, immune cell populations, extracellular matrix components, and vascular networks that actively support tumor growth, invasion, and metastatic dissemination [3,4,5]. A pivotal role is played by the invasive tumor front (ITF), which represents the interface between malignant and adjacent normal tissue and serves as a key site where the biological processes underlying tumor progression are concentrated. At this boundary, epithelial–mesenchymal transition (EMT), extracellular matrix remodeling, immune modulation, and angiogenesis interact in a coordinated manner to enhance tumor invasiveness [1,6,7,8,9,10,11,12].

EMT-associated features are particularly prominent at the invasive margin and are closely linked to stromal activation, including the accumulation of cancer-associated fibroblasts (CAFs) and the activation of signaling pathways such as TGF-β and Wnt [13,14,15,16]. In this context, tumor budding has emerged as a strong adverse histopathological indicator and is widely regarded as a morphological correlate of EMT [4,17,18]. However, tumor invasion reflects not only epithelial plasticity but also a complex interplay with the surrounding microenvironment [13,17,19,20]. Stromal components, particularly CAFs, actively remodel the extracellular matrix and influence tumor behavior, while immune populations such as tumor-associated macrophages (TAMs) contribute to a permissive microenvironment through immunosuppressive and pro-angiogenic functions [14,21,22,23,24]. Angiogenesis represents another crucial component of tumor progression and is tightly integrated with stromal remodeling and immune regulation [25,26,27,28,29]. In addition, mast cells are increasingly recognized as important regulators of the TME through the release of cytokines, proteases, and pro-angiogenic mediators [30,31,32,33].

Taken together, these observations support the concept that the ITF should be interpreted as a biologically integrated compartment rather than as a site defined solely by epithelial dissociation. On this basis, the aim of the present exploratory study was to investigate whether a combined histopathological evaluation of tumor budding and selected stromal, immune, and vascular markers at the ITF could provide a broader characterization of aggressive clinicopathological features in non-metastatic CRC and whether these microenvironment-related variables could be incorporated into an integrated histopathological association score of the invasive front. This study was not designed to establish prognostic value or molecular correlations.

## 2. Results

### 2.1. Patient Characteristics

The clinicopathological characteristics of the 52 non-metastatic CRC patients included in this study are summarized in Table 1. Patients with distant metastatic disease at diagnosis or at surgery (M+) were excluded.

The mean age was 74 ± 11.9 years; 24 patients (46.2%) were younger than 74 years and 28 (53.8%) were aged 74 years or older. The cohort was equally distributed by sex, with 26 male patients (50.0%) and 26 female patients (50.0%).

Thirty-six tumors (69.2%) were classified as low grade and 16 (30.8%) as high grade. According to nodal status, 18 cases (34.6%) were node-negative (N0), whereas 34 (65.4%) were node-positive (N+). Histologically, 38 tumors (73.1%) were classified as conventional adenocarcinomas, whereas 14 (26.9%) were classified as unconventional histological subtypes according to standardized histopathological criteria, including 9 mucinous adenocarcinomas, 2 diffuse/signet-ring cell carcinomas, and 3 cribriform carcinomas. Because of the limited number of cases in each individual subtype, these variants were grouped as unconventional subtypes for exploratory statistical purposes. Lymphovascular invasion was absent in 18 cases (34.6%) and present in 34 (65.4%). Low tumor budding was observed in 28 cases (53.8%), whereas 24 cases (46.2%) showed high tumor budding.

**Figure 1:**
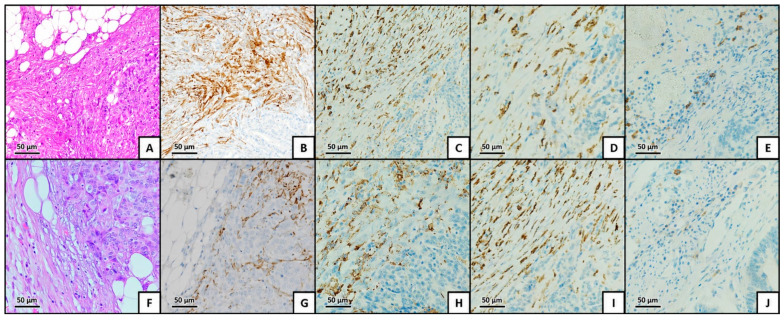
Representative high-power fields at the invasive tumor front. Upper row: case with high tumor budding: (**A**) hematoxylin and eosin (H&E) staining, 400×; (**B**) podoplanin immunohistochemical staining, 400×; (**C**) CD163 immunohistochemical staining, 400×; (**D**) CD68 immunohistochemical staining, 400×; (**E**) CD117 immunohistochemical staining, 400×. Lower row: case with low tumor budding: (**F**) hematoxylin and eosin (H&E) staining, 400×; (**G**) podoplanin immunohistochemical staining, 400×; (**H**) CD163 immunohistochemical staining, 400×; (**I**) CD68 immunohistochemical staining, 400×; (**J**) CD117 immunohistochemical staining, 400×. Scale bars are included in each panel.

Regarding microenvironment-related markers, the CD163/CD68 ratio was low in 29 cases (55.8%) and high in 23 (44.2%). Low D2-40 stromal expression was observed in 36 cases (69.2%), whereas high expression was found in 16 (30.8%). In addition to stromal staining, podoplanin expression was also observed in neoplastic cells in 10 cases, showing strong membranous positivity. Representative histological and immunohistochemical images of high- and low-budding cases at the invasive tumor front are shown in Figure 1. CD117-positive mast cell density was low in 33 cases (63.5%) and high in 19 (36.5%). Finally, MVD was low in 32 cases (61.5%) and high in 20 (38.5%).

When the four dichotomized microenvironment-related markers were integrated into a composite ITF microenvironment score, 24 cases (46.2%) had a score of 0, 8 (15.4%) a score of 1, 2 (3.8%) a score of 2, 6 (11.5%) a score of 3, and 12 (23.1%) a score of 4.

### 2.2. Correlation Between Clinicopathological and Microenvironment-Related Parameters

The exploratory pairwise associations between clinicopathological features and TME-related markers are summarized in Table 2. Because multiple comparisons were performed, the unadjusted *p* values shown in Table 2 were further assessed using a Benjamini–Hochberg FDR sensitivity analysis, reported in Supplementary Table S1. Associations retaining statistical significance after FDR correction are indicated in Supplementary Table S1.

No significant associations were observed between age or sex and the analyzed pathological or microenvironmental variables, although a borderline association between age category and the CD163/CD68 ratio was noted (*p* = 0.054; OR 3.24, 95% CI 0.98–10.28).

Histological grade showed significant associations with established adverse pathological features. High-grade tumors were significantly associated with lymphovascular invasion (*p* = 0.004; OR 13.42, 95% CI 1.6–112.6), high tumor budding (*p* = 0.001; OR 9.84, 95% CI 2.3–41.6), and positive nodal status (*p* = 0.004; OR 13.42, 95% CI 1.6–112.6). High grade was also significantly associated with all evaluated microenvironment-related parameters, including a high CD163/CD68 ratio (*p* = 0.006; OR 6.8, 95% CI 1.8–25.9), high D2-40 stromal expression (*p* < 0.0001; OR 24, 95% CI 5.16–111.6), high CD117-positive mast cell density (*p* < 0.0001; OR 43.4, 95% CI 7.5–251.5), and high MVD (*p* < 0.0001; OR 35, 95% CI 6.26–195.7).

Histological subtype was not significantly associated with most of the analyzed variables, although a trend toward association with D2-40 stromal expression was observed (*p* = 0.09). Lymphovascular invasion was significantly associated with high tumor budding (*p* < 0.0001; OR 35.55, 95% CI 4.18–302.4), positive nodal status (*p* = 0.0057; OR 6.06, 95% CI 1.72–21.38), a high CD163/CD68 ratio (*p* = 0.038; OR 4.43, 95% CI 1.21–16.29), high D2-40 stromal expression (*p* = 0.0044; OR 13.42, 95% CI 1.6–112.64), high CD117-positive mast cell density (*p* = 0.0007; OR 19.12, 95% CI 2.28–160.33), and high MVD (*p* < 0.0001; OR infinite, 95% CI 2.85–927.08).

Tumor budding was significantly associated with positive nodal status (*p* = 0.003; OR 8.07, 95% CI 1.95–33.4), a high CD163/CD68 ratio (*p* = 0.0006; OR 8.9, 95% CI 2.52–31.42), high D2-40 stromal expression (*p* < 0.0001; OR 45, 95% CI 5.18–390.3), high CD117-positive mast cell density (*p* < 0.0001; OR 81, 95% CI 8.98–730.6), and high MVD (*p* < 0.0001; OR 102.6, 95% CI 11.08–950.2).

Similarly, node-positive disease was significantly associated with a high CD163/CD68 ratio (*p* = 0.0005; OR 12.92, 95% CI 2.54–65.6), high D2-40 stromal expression (*p* = 0.0044; OR 13.42, 95% CI 1.59–112.6), high CD117-positive mast cell density (*p* = 0.0007; OR 19.13, 95% CI 2.28–160.3), and high MVD (*p* = 0.006; OR 9, 95% CI 1.78–45.34).

Significant associations were also observed among microenvironment-related parameters. A high CD163/CD68 ratio was associated with high D2-40 stromal expression (*p* = 0.0006; OR 11.27, 95% CI 2.63–48.12) and high CD117-positive mast cell density (*p* = 0.0002; OR 11.72, 95% CI 3–45.67). High D2-40 stromal expression was associated with high CD117-positive mast cell density (*p* < 0.0001; OR infinite, 95% CI 16.62–7454) and high MVD (*p* < 0.0001; OR 93, 95% CI 9.96–868.16). High CD117-positive mast cell density was, in turn, associated with high MVD (*p* < 0.0001; OR 279, 95% CI 23.61–3298).

Infinite OR values occurred in comparisons with zero cells in contingency tables and should not be interpreted as precise estimates of effect magnitude. They are reported for transparency and interpreted only as exploratory indicators of strong association in sparse data.

Overall, these findings support the presence of coordinated histopathological remodeling at the invasive tumor front. However, some pairwise comparisons yielded very large ORs with wide confidence intervals, reflecting the limited sample size and the presence of sparse contingency tables. Accordingly, these effect-size estimates should be interpreted cautiously, and greater emphasis should be placed on the overall pattern of association than on the precise magnitude of individual OR values.

The interobserver reliability among pathologists for all analyzed parameters was in the range of almost perfect agreement, ranging from Cohen’s kappa 0.85 to 0.92.

### 2.3. Integrated ITF Microenvironment Score

To explore whether the selected stromal, immune, and vascular parameters could be synthesized into a composite histopathological association indicator, we generated an integrated ITF microenvironment score by summing four dichotomized variables: high D2-40 stromal expression, high CD163/CD68 ratio, high CD117-positive mast cell density, and high MVD. The resulting score ranged from 0 to 4.

The integrated ITF microenvironment score was significantly higher in tumors with adverse clinicopathological features. In particular, high-grade tumors showed a significantly higher score than low-grade tumors (median 3.5, IQR 3.0–4.0 vs. median 0.0, IQR 0.0–1.0; *p* < 0.0001). Likewise, tumors with lymphovascular invasion had a higher score than those without lymphovascular invasion (median 2.5, IQR 0.0–4.0 vs. median 0.0, IQR 0.0–0.0; *p* = 0.0003). Node-positive tumors also showed significantly higher scores than node-negative tumors (median 2.5, IQR 0.25–4.0 vs. median 0.0, IQR 0.0–0.0; *p* = 0.0001). Finally, tumors with high budding activity displayed markedly higher scores than low-budding tumors (median 3.5, IQR 2.0–4.0 vs. median 0.0, IQR 0.0–0.25; *p* < 0.0001).

Overall, these findings indicate that adverse pathological features were not associated with isolated alterations in single markers, but rather with the progressive accumulation of coordinated stromal, immune, and vascular changes at the invasive front.

## 3. Discussion

In the present series, adverse clinicopathological features of colorectal carcinoma were accompanied by coordinated changes in stromal, immune, and vascular markers at the ITF. In particular, high histological grade, lymphovascular invasion, tumor budding, and positive nodal status were consistently associated with increased D2-40 stromal expression, a higher CD163/CD68 ratio, increased CD117-positive mast cell density, and increased MVD. These findings support the concept that the ITF is not merely a site of epithelial dissociation, but rather a biologically integrated compartment in which epithelial, stromal, immune, and vascular processes converge.

ITF represents a biologically active and highly dynamic compartment in colorectal carcinoma, where tumor cells and the surrounding microenvironment interact in a coordinated manner to drive disease progression [2,3,4,5]. Tumor budding, a well-established adverse histopathological feature, reflects partial epithelial–mesenchymal transition, a dynamic and reversible process characterized by hybrid epithelial–mesenchymal phenotypes [1,6,7,8]. EMT is strongly influenced by microenvironmental signals, including cytokines and growth factors, thereby reinforcing tumor invasiveness through bidirectional tumor-stroma crosstalk [5,17,19]. The stromal compartment is characterized by the presence of cancer-associated fibroblasts, a heterogeneous population involved in extracellular matrix remodeling and tumor progression [10,13,14,15,16,34]. In our cohort, high D2-40 stromal expression was significantly associated with high histological grade, lymphovascular invasion, tumor budding, positive nodal status, CD117-positive mast cell density, and MVD [35,36,37]. Although podoplanin is not exclusively restricted to CAFs, these findings are consistent with the interpretation that stromal activation is a relevant component of aggressive tumor behavior at the invasive front. The immune component showed a similarly coordinated pattern. TAMs, particularly CD163-enriched populations, are known to promote tumor progression, EMT, and immune evasion through the release of cytokines and growth factors [14,21,22,23,30,38]. In the present study, a high CD163/CD68 ratio, used as a surrogate of macrophage polarization, was significantly associated with high grade, lymphovascular invasion, tumor budding, and positive nodal status. These data further support the view that macrophage polarization is closely integrated with adverse histopathological remodeling at the invasive front. Angiogenesis, reflected by increased MVD, represents another key component of tumor progression and is tightly connected to immune regulation and stromal remodeling [24,25,26,27]. In our series, high MVD was significantly associated with grade, lymphovascular invasion, tumor budding, positive nodal status, D2-40 stromal expression, and CD117-positive mast cell density. Likewise, mast cells are increasingly recognized as important regulators of the TME through the release of cytokines, proteases, and pro-angiogenic mediators [30,31,32,33,36]. The strong associations observed between CD117-positive mast cell density, D2-40 stromal expression, and MVD are in keeping with a coordinated microenvironmental network rather than isolated alterations affecting single cellular compartments.

At this stage, the integrated ITF microenvironment score was intentionally constructed as an unweighted additive score, with each parameter contributing one point. This choice was made for both biological and statistical reasons. Biologically, D2-40 stromal expression, CD163/CD68 ratio, CD117-positive mast cell density, and MVD represent distinct but interdependent compartments of the invasive-front microenvironment, namely stromal activation, macrophage polarization, mast-cell enrichment, and angiogenic remodeling. Statistically, the present cohort was not sufficiently powered to generate or validate differential weights for individual variables. Although some markers, particularly CD117-positive mast cell density, D2-40 stromal expression, and MVD, showed numerically stronger associations in selected pairwise analyses, these estimates were affected by sparse tables and wide confidence intervals. Therefore, assigning greater weight to one marker would risk overfitting and would imply a degree of validation not supported by the present exploratory design [39]. For these reasons, all four ITF parameters were considered to have equal weight in the current score. Future larger, outcome-annotated cohorts should investigate whether a weighted score, derived from multivariable regression or survival models, provides superior prognostic performance compared with the present unweighted approach.

An additional strength of the present study is that these coordinated changes could also be synthesized into a composite ITF microenvironment score. This integrated score increased significantly in association with high histological grade, lymphovascular invasion, nodal involvement, and high tumor budding, suggesting that aggressive colorectal carcinomas are characterized not simply by one adverse feature at the invasive front, but by the cumulative activation of multiple stromal, immune, and vascular compartments. From a conceptual standpoint, this score may better reflect the biological complexity of the invasive front than any single marker considered in isolation. Importantly, however, the score should not currently be interpreted as a weighted prognostic model, but rather as a pragmatic exploratory measure of cumulative microenvironmental burden. In this respect, the integrated score is consistent with the central hypothesis of the present study, namely that tumor budding should be interpreted within a broader microenvironmental context rather than as an isolated epithelial phenomenon.

From a practical standpoint, the evaluation of the ITF relies on routinely available histopathological and immunohistochemical parameters, making this approach potentially applicable in diagnostic practice. In this setting, the present findings suggest that tumor budding may reflect only one component of a broader biological program operating at the invasive front, where epithelial detachment is accompanied by stromal activation, macrophage polarization, mast cell enrichment, and angiogenic remodeling [12,28,29].

A relevant limitation of the present study is the absence of molecular tumor annotation. We fully agree that the integration of APC, TP53, KRAS, NRAS, BRAF, SMAD4 and MMR/MSI status would provide important biological context for interpreting ITF microenvironmental remodeling. However, the present study was designed and approved as a retrospective histopathological and immunohistochemical analysis of invasive-front features, and molecular testing was not performed as part of the original analytical workflow. At this revision stage, complete molecular characterization of all 52 cases would require a dedicated additional workflow, including tissue selection, nucleic-acid extraction and quality control, validated molecular assays, and harmonized interpretation. Performing incomplete or subset-based molecular testing would reduce the cohort size, introduce selection bias, and generate underpowered subgroup analyses. Therefore, molecular correlations were not added to the current manuscript. Future studies will specifically address the relationship between the integrated ITF microenvironment score and CRC molecular alterations, including APC, TP53, KRAS, NRAS, BRAF, SMAD4 and MMR/MSI status.

Several statistical and clinicopathological considerations should be taken into account when interpreting the present results. First, this study was based on a relatively limited sample size, which increases the instability of effect-size estimates in subgroup analyses. Second, the analysis relied on multiple pairwise comparisons between dichotomized variables and did not include multivariable modeling; therefore, it was not designed to establish independence or causality. Third, some associations were characterized by sparse contingency tables, which may generate very large or infinite OR estimates and wide confidence intervals. In this context, the ORs reported in the present study should be interpreted primarily as exploratory indicators of association strength rather than as precise quantitative measures of effect magnitude. Fourth, although a Benjamini–Hochberg FDR sensitivity analysis was added in Supplementary Table S1, the results remain exploratory and hypothesis generating. The Supplementary Table reports each pairwise comparison, the unadjusted *p* value, the corresponding FDR-adjusted q value, and whether the association remained significant after FDR correction. Fifth, standardized follow-up data suitable for OS, DFS, RFS, disease-specific survival, or recurrence analysis were not available for the entire cohort. Several patients were not treated or followed after surgery at our institution, adjuvant and subsequent therapy information was incomplete, and systematic patient re-contact was not feasible within the retrospective anonymized design of this study. Therefore, no direct conclusions can be drawn regarding the prognostic value of the evaluated markers or of the integrated ITF microenvironment score. Sixth, molecular tumor data, including APC, TP53, KRAS, NRAS, BRAF, SMAD4 and MMR/MSI status, were not available because they were not generated in the original study workflow. Seventh, tumor location, complete pT category, complete TNM stage group, MMR/MSI status, RAS/BRAF mutational status, bowel obstruction or perforation, adjuvant therapy, and standardized follow-up/recurrence data were not uniformly available in the anonymized analytical dataset. Finally, the integrated score proposed here should be regarded as exploratory and hypothesis generating. No differential weighting of individual ITF parameters was attempted, because this would require larger datasets and external validation. Additional studies in larger, independent, molecularly annotated and outcome-annotated cohorts will be necessary to determine its reproducibility, optimal categorization, potential weighting, and prognostic or predictive relevance. Accordingly, the present findings should be interpreted as a histopathological framework for subsequent validation studies rather than as evidence for immediate clinical implementation.

## 4. Materials and Methods

### 4.1. Patients and Clinicopathological Data

This retrospective study included 52 patients who underwent surgical resection for primary non-metastatic colorectal adenocarcinoma at A.O.U. Policlinico “G. Martino”, Messina, Italy, between January 2021 and October 2023. Patients with distant metastatic disease at diagnosis or at surgery (M+) were excluded. Surgical specimens were fixed in 10% neutral buffered formalin (pH 7.0) for 24–48 h and embedded in paraffin. Sections 4–5 μm thick were stained with hematoxylin and eosin for routine histopathological evaluation.

Tumors were pathologically classified according to the AJCC 8th edition criteria when available in the pathology records. For the purposes of the present correlation analysis, no early-stage versus advanced-stage grouping was generated or used. The clinicopathological variable entered into the statistical model was nodal status, dichotomized as N0 versus N+. The M category was used only to define cohort eligibility. Cases without evidence of distant metastatic disease at diagnosis or at surgery (M0) were included, whereas cases with distant metastatic disease (M+/M1) were excluded. This criterion was adopted to obtain a more homogeneous cohort of surgically resected primary non-metastatic CRCs and to avoid the confounding effect of overt systemic metastatic disease on the interpretation of invasive-front microenvironmental features. Therefore, the present analyses were focused on pathological associations within the primary tumor rather than on metastatic disease biology or prognosis.

Patients were eligible for inclusion if they had: (1) histologically confirmed primary colorectal adenocarcinoma; (2) surgical resection with available formalin-fixed, paraffin-embedded tissue suitable for histopathological and immunohistochemical evaluation; (3) adequate representation of the ITF on histological sections; (4) no evidence of distant metastatic disease at diagnosis or at surgery (M0); and (5) complete clinicopathological data required for the planned pathological association analyses. Exclusion criteria were: (1) recurrent colorectal cancer; (2) synchronous or metachronous colorectal tumors; (3) neoadjuvant treatment; (4) distant metastatic disease at diagnosis or at surgery (M+); (5) insufficient tissue for reliable evaluation; (6) absent or poorly represented ITF; and (7) incomplete clinical or pathological data. Histological subtype was assigned on hematoxylin and eosin-stained sections according to the World Health Organization classification criteria for colorectal carcinoma. Tumors were categorized as conventional adenocarcinoma or unconventional histological subtype. The unconventional group included mucinous adenocarcinoma, diffuse/signet-ring cell carcinoma, and cribriform carcinoma. This grouping was used only for exploratory statistical purposes because of the limited number of cases in each individual subtype. Histological grade was dichotomized as low grade and high grade for statistical purposes; low grade included well and moderately differentiated tumors, whereas high grade included poorly differentiated tumors. Molecular analyses, including APC, TP53, KRAS, NRAS, BRAF, SMAD4 mutational status and MMR/MSI assessment, were not performed as part of the original study workflow and were therefore not available for systematic correlation with the immunohistochemical variables. For this reason, molecular variables were not included in the statistical analyses. Tumor location, complete pT category, complete TNM stage group, MMR/MSI status, RAS/BRAF mutational status, bowel obstruction or perforation, adjuvant therapy, and standardized follow-up endpoints were not uniformly available in the anonymized analytical dataset and were therefore not included in the association analyses. In particular, several patients were not treated or followed after surgery at the same institution, information on adjuvant or subsequent therapies was incomplete, and systematic patient re-contact was not feasible within the retrospective anonymized study design. Consequently, standardized follow-up endpoints suitable for OS, DFS, RFS, disease-specific survival, or recurrence analysis could not be reliably reconstructed. No early-stage versus advanced-stage grouping was generated.

All data were collected and analyzed anonymously. This study was conducted in accordance with the Declaration of Helsinki and approved by the Local Ethics Committee of Messina AOU G. Martino (protocol code PNRR-MCNT1-2023-12378355, approved on 22 July 2024). The approval specifically covered the retrospective analysis of archived histological material and anonymized clinicopathological data. Before surgery/admission, all patients had signed broad written informed consent authorizing the storage and scientific use of residual histological material for research purposes. Therefore, although the cases were collected between January 2021 and October 2023, informed consent for research use of the samples had already been obtained before the retrospective study approval.

### 4.2. Immunohistochemistry for D2-40, CD68, CD163, CD34, and CD117

Immunohistochemical analyses were performed on formalin-fixed, paraffin-embedded tissue samples. Serial sections (3 μm thick) were cut using a microtome, mounted on positively charged slides (Superfrost Plus, Thermo Fisher Scientific, Waltham, MA, USA), and dried at 60 °C for 1 h. Immunostaining was carried out using an automated staining system (Ventana BenchMark ULTRA, Roche Diagnostics, Tucson, AZ, USA) according to the manufacturer’s instructions, with deparaffinization performed on-board.

Heat-induced epitope retrieval was conducted using Cell Conditioning 1 (CC1; Tris–EDTA buffer, pH 8.0–8.5) or Cell Conditioning 2 (CC2; citrate buffer, pH approximately 6.0), depending on the antibody, with incubation times ranging from 36 to 64 min at 95–100 °C. The following primary antibodies were used: anti-podoplanin (D2-40, clone D2-40), anti-CD68 (clone KP1), anti-CD163 (clone MRQ-26), anti-CD34 (clone QBEnd/10), and anti-CD117 (c-KIT, clone YR145), all in ready-to-use formulations (Roche/Ventana). Primary antibody incubation was performed automatically for 16–32 min at 37 °C. Immunoreactivity was visualized using a polymer-based detection system with horseradish peroxidase (ultraView Universal DAB Detection Kit, Roche/Ventana), with 3,3′-diaminobenzidine (DAB) as the chromogen. Slides were counterstained with Mayer’s hematoxylin, followed by bluing, dehydration through graded alcohols, clearing in xylene, and mounting with a permanent medium. Appropriate positive controls and negative controls were included in each staining run.

For all markers, the invasive tumor front (ITF) was identified on the corresponding hematoxylin and eosin-stained section and subsequently matched with the immunostained slides. The ITF was defined as the deepest advancing edge of the tumor, corresponding to the interface between neoplastic glands or cells and the adjacent non-neoplastic tissue. Areas affected by major tissue folds, necrosis, extensive crush artifact, cautery artifact, or poor staining preservation were excluded from evaluation. All assessments were performed on whole-tissue sections.

### 4.3. Evaluation of ITF Parameters

Tumor budding was assessed at the ITF according to the criteria established by the International Tumor Budding Consensus Conference (ITBCC) 2016 [4]. After scanning the invasive margin at low magnification to identify the hotspot, tumor buds were counted in a single hotspot field measuring 0.785 mm^2^ using a 20× objective lens. A tumor bud was defined as a single tumor cell or a cluster of up to four tumor cells. Cases were categorized as BD1, BD2, and BD3, corresponding to low, intermediate, and high budding activity, respectively [4,18]. For statistical analysis, cases were grouped as low tumor budding (BD1) and high tumor budding (BD2–BD3).

To improve reproducibility across the different ITF parameters, the number of fields evaluated was specified a priori. Tumor budding was assessed in one ITBCC hotspot field measuring 0.785 mm^2^ using a 20× objective lens [4]. For immunohistochemical ITF parameters, the entire invasive front was first scanned at low magnification to identify the most representative or highest-density areas, after which non-overlapping high-power fields (HPFs) were evaluated at 400× magnification. D2-40 stromal expression was assessed in five representative HPFs at the ITF and the final score was assigned according to the overall percentage of positive stromal cells in these fields. CD34-based MVD was assessed in the three most vascular HPFs at the ITF, and the mean microvessel count was used for analysis [40]. CD68-positive and CD163-positive macrophages were counted in five corresponding HPFs at the ITF, and the CD163/CD68 ratio was calculated from the mean counts [41]. CD117-positive mast cells were evaluated in five hotspot HPFs showing the highest mast-cell density.

Podoplanin (D2-40) stromal expression at the ITF was semiquantitatively assessed in the stromal compartment immediately adjacent to the invasive front according to previously published approaches [10,35,42]. Particular attention was paid to stromal spindle-shaped cells morphologically consistent with activated fibroblastic elements, while endothelial lymphatic staining served as an internal positive control and was not included in the stromal score. After low-power scanning of the entire invasive front, five representative non-overlapping HPFs (400×) were selected for semiquantitative evaluation. Stromal staining was scored as 0 (absent or weak staining in <1% of tumor stroma), 1 (focal positivity in 1–10% of stromal cells), 2 (positivity in 11–50% of stromal cells), and 3 (positivity in 51–100% of stromal cells). For statistical purposes, D2-40 stromal expression was dichotomized into low (scores 0–2) and high (score 3). When present, neoplastic cell staining was recorded descriptively but was not included in stromal scoring.

Microvessel density (MVD) was determined at the ITF on CD34-immunostained sections. After low-power scanning to identify the areas with the highest degree of vascularization, microvessels were counted in the three most vascular non-overlapping HPFs (400×) at the invasive front, and the mean microvessel count was used for analysis. Any brown-stained endothelial cell or endothelial cell cluster clearly separated from adjacent vessels, tumor cells, or other connective tissue elements was considered a countable microvessel, irrespective of the presence of a visible lumen [10,40,41]. Large vessels with thick muscular walls were excluded. Cases were subsequently stratified into low and high MVD groups according to the cohort mean value.

Tumor-associated macrophages (TAMs) were assessed at the ITF using CD68 and CD163 immunostaining. The invasive front was first scanned at low magnification to identify representative hotspot areas with the highest density of positively stained stromal inflammatory cells. In five corresponding non-overlapping HPFs (400×), only cells showing appropriate macrophage morphology and unequivocal cytoplasmic immunoreactivity were counted, whereas intraluminal cells and nonspecific background staining were excluded. For each case, mean CD68-positive and CD163-positive cell counts were calculated across the evaluated fields, and the CD163/CD68 ratio was used as a surrogate of macrophage polarization. For statistical purposes, cases were classified as low or high according to the predefined cut-off value of 1.38.

CD117-positive mast cells were evaluated at the ITF in five non-overlapping hotspot HPFs (400×) showing the highest mast cell density at low magnification. Only cells with positive cytoplasmic staining and compatible mast-cell morphology were considered [9,36,43]. Particular attention was paid to the characteristic size, shape, and granular cytoplasmic pattern of mast cells in order to avoid misinterpretation of other CD117-positive elements. CD117-positive mast cell density was semiquantitatively scored as 0, absent; 1, rare/scattered positive mast cells; 2, moderate accumulation; and 3, dense clustered accumulation in hotspot HPFs. For descriptive and statistical purposes, cases were categorized as low (scores 0–1) or high (scores 2–3) CD117-positive mast cell density [9,36,43].

All histopathological and immunohistochemical assessments were independently performed by two experienced pathologists blinded to clinicopathological data. In cases of initial disagreement, the slides were jointly reviewed at a multiheaded microscope and a final consensus score was assigned. To evaluate interobserver agreement in morphological and immunohistochemical interpretation, Cohen’s kappa was applied.

### 4.4. Statistical Analysis

Statistical analyses were performed using GraphPad Prism 5 (GraphPad Software, San Diego, CA, USA) and MedCalc version 10.2.0.0 (MedCalc Software, Mariakerke, Belgium). Continuous variables were expressed as mean ± standard deviation (SD), whereas categorical variables were reported as number and percentage.

For statistical analysis, variables were dichotomized as follows: age (<74 vs. ≥74 years), histological grade (low vs. high), nodal status (N0 vs. N+), histological subtype (conventional vs. unconventional), lymphovascular invasion (absent vs. present), tumor budding (low [BD1] vs. high [BD2–BD3]), D2-40 stromal expression (low [scores 0–2] vs. high [score 3]), CD117-positive mast cell density (low [scores 0–1] vs. high [scores 2–3]), MVD (low vs. high according to the cohort mean value), and CD163/CD68 ratio (low vs. high according to the predefined cut-off of 1.38). No early-stage versus advanced-stage grouping was generated. The dichotomization strategy was chosen to reduce sparse categories in this exploratory cohort and to allow for pairwise association analyses across clinicopathological and ITF-related variables. The D2-40 score 3 cut-off was selected to identify cases with diffuse stromal activation; CD117 scores 2–3 were grouped to identify cases with moderate-to-high mast cell enrichment; MVD was dichotomized using the cohort mean because no universally accepted ITF-specific threshold is available; and the CD163/CD68 ratio cut-off of 1.38 was retained as a predefined threshold for macrophage polarization at the invasive tumor front.

In addition, an exploratory unweighted integrated ITF microenvironment score ranging from 0 to 4 was generated by summing four dichotomized microenvironment-related parameters: high D2-40 stromal expression, high CD163/CD68 ratio, high CD117-positive mast cell density, and high MVD. Thus, each case received one point for each adverse microenvironment-related feature present at the invasive front. All four variables were assigned equal weight because this study was exploratory and was not powered to derive or validate differential weighting coefficients [39].

Associations between categorical variables were analyzed using the chi-square test or Fisher’s exact test, as appropriate. Fisher’s exact test was preferred whenever contingency tables were sparse or expected cell counts were low. Odds ratios (ORs) with 95% confidence intervals (95% CIs) were calculated for pairwise associations between dichotomized variables and were interpreted as exploratory measures of effect size. The integrated ITF microenvironment score was analyzed as an ordinal variable, and between-group comparisons were performed using the Mann–Whitney U test. Given the limited sample size and the presence of sparse tables in some comparisons, OR estimates associated with very wide confidence intervals were interpreted cautiously. Because of the exploratory nature of this study, unadjusted *p* values are reported in Table 2. In response to the risk of type I error related to multiple pairwise comparisons, a supplementary sensitivity analysis using the Benjamini–Hochberg false discovery rate (FDR) procedure was also performed and is reported in Supplementary Table S1. Therefore, the statistical analysis should be regarded as exploratory and hypothesis generating rather than confirmatory. The interobserver agreement of pathologists in morphological and immunohistochemical interpretation was determined via Cohen’s kappa with 95% confidence intervals [44]. A *p* value < 0.05 was considered statistically significant. All tests were two-sided.

## 5. Conclusions

In conclusion, the ITF represents a biologically relevant compartment in non-metastatic colorectal carcinoma in which epithelial, stromal, immune, and vascular changes appear to be closely interconnected. In this exploratory histopathological association study, an integrated assessment of the invasive front provided a broader overview of tumor-associated microenvironmental remodeling than tumor budding alone. The composite ITF microenvironment score supported the concept that adverse pathological features are associated with the accumulation of coordinated stromal, immune, and vascular alterations at the invasive front. However, the score should not be interpreted as a validated prognostic model. These findings require confirmation in larger, independent, molecularly annotated and outcome-annotated cohorts before any clinical application can be proposed.

## Figures and Tables

**Table 1:** Clinicopathological characteristics of the study cohort.

Variable	n = 52
Age, mean ± SD	74 ± 11.9
Age, n (%)	
<74	24 (46.2%)
≥74	28 (53.8%)
Sex, n (%)	
Male	26 (50.0%)
Female	26 (50.0%)
Histological grade, n (%)	
Low	36 (69.2%)
High	16 (30.8%)
Nodal status, n (%)	
N0	18 (34.6%)
N+	34 (65.4%)
Histological subtype, n (%)	
Conventional	38 (73.1%)
Unconventional	14 (26.9%)
Lymphovascular invasion, n (%)	
Absent	18 (34.6%)
Present	34 (65.4%)
Tumor budding, n (%)	
Low (BD1)	28 (53.8%)
High (BD2–BD3)	24 (46.2%)
CD163/CD68 ratio, n (%)	
Low	29 (55.8%)
High	23 (44.2%)
D2-40 stromal expression, n (%)	
Low (scores 0–2)	36 (69.2%)
High (score 3)	16 (30.8%)
CD117-positive mast cell density, n (%)	
Low (scores 0–1)	33 (63.5%)
High (scores 2–3)	19 (36.5%)
MVD, n (%)	
Low	32 (61.5%)
High	20 (38.5%)
Integrated ITF microenvironment score, n (%)	
0	24 (46.2%)
1	8 (15.4%)
2	2 (3.8%)
3	6 (11.5%)
4	12 (23.1%)

Abbreviations: ITF, invasive tumor front; MVD, microvessel density; SD, standard deviation. Patients with distant metastatic disease at diagnosis or at surgery (M+) were excluded.

**Table 2:** Pairwise associations between clinicopathological features and microenvironment-related parameters.

	Sex (Male vs. Female)	Grade (Low vs. High)	Histological SUBTYPE (Unconventional vs. Conventional)	Lymphovascular Invasion (Absent vs. Present)	Tumor Budding (Low vs. High)	Nodal Status (N0 vs. N+)	CD163/CD68 Ratio (Low vs. High)	D2-40 Stromal Expression (Low vs. High)	CD117-Positive Mast Cell Density (Low vs. High)	MVD (Low vs. High)
	*p*, OR (95% CI)	*p*, OR (95% CI)	*p*, OR (95% CI)	*p*, OR (95% CI)	*p*, OR (95% CI)	*p*, OR (95% CI)	*p*, OR (95% CI)	*p*, OR (95% CI)	*p*, OR (95% CI)	*p*, OR (95% CI)
Age (<74 vs. ≥74)	0.78, 1.36 (95% CI 0.46 to 4.07)	0.77, 0.8 (95% CI 0.25 to 2.6)	0.76, 1.24 (95% CI 0.36 to 4.22)	0.77, 1.27 (95% CI 0.4 to 3.98)	0.28, 1.92 (95% CI 0.63 to 5.84)	0.77, 1.27 (95% CI 0.4 to 3.98)	0.054, 3.24 (95% CI 0.98 to 10.28)	0.55, 1.67 (95% CI 0.5 to 5.56)	0.78, 1.29 (95% CI 0.41 to 4.04)	0.57, 1.5 (95% CI 0.48 to 4.65)
Sex (Male vs. Female)	—	0.37, 0.48 (95% CI 0.14 to 1.6)	0.35, 0.45 (95% CI 0.13 to 1.6)	0.38, 0.5 (95% CI 0.16 to 1.61)	0.78, 0.73 (95% CI 0.25 to 2.19)	0.38, 0.5 (95% CI 0.16 to 1.61)	0.26, 0.45 (95% CI 0.15 to 1.39)	0.37, 0.48 (95% CI 0.14 to 1.6)	0.57, 0.61 (95% CI 0.19 to 1.89)	0.78, 0.72 (95% CI 0.24 to 2.22)
Grade (Low vs. High)	—	—	0.32, 0.48 (95% CI 0.13 to 1.71)	0.0044, 13.42 (95% CI 1.6 to 112.64)	0.001, 9.85 (95% CI 2.33 to 41.64)	0.0044, 13.42 (95% CI 1.6 to 112.64)	0.0056, 6.82 (95% CI 1.79 to 25.92)	<0.0001, 24 (95% CI 5.16 to 111.56)	<0.0001, 43.4 (95% CI 7.49 to 251.52)	<0.0001, 35 (95% CI 6.26 to 195.74)
Histological subtype (Unconventional vs. Conventional)	—	—	—	1, 1.07 (95% CI 0.3 to 3.85)	0.13, 0.36 (95% CI 0.1 to 1.29)	0.33, 0.42 (95% CI 0.1 to 1.75)	0.76, 0.73 (95% CI 0.21 to 2.49)	0.094, 0.31 (95% CI 0.09 to 1.12)	0.33, 0.46 (95% CI 0.13 to 1.61)	0.12, 0.35 (95% CI 0.1 to 1.22)
Lymphovascular invasion (Absent vs. Present)	—	—	—	—	<0.0001, 35.55 (95% CI 4.18 to 302.4)	0.0057, 6.06 (95% CI 1.72 to 21.38)	0.038, 4.43 (95% CI 1.21 to 16.29)	0.0044, 13.42 (95% CI 1.6 to 112.64)	0.0007, 19.12 (95% CI 2.28 to 160.33)	<0.0001, infinite (95% CI 2.85 to 927.08)
Tumor budding (Low vs. High)	—	—	—	—	—	0.003, 8.08 (95% CI 1.95 to 33.4)	0.0006, 8.9 (95% CI 2.52 to 31.42)	<0.0001, 45 (95% CI 5.19 to 390.3)	<0.0001, 81 (95% CI 8.98 to 730.59)	<0.0001, 102.6 (95% CI 11.08 to 950.17)
Nodal status (N0 vs. N+)	—	—	—	—	—	—	0.0005, 12.92 (95% CI 2.55 to 65.6)	0.0044, 13.42 (95% CI 1.6 to 112.64)	0.0007, 19.12 (95% CI 2.28 to 160.33)	0.006, 9 (95% CI 1.79 to 45.34)
CD163/CD68 ratio (Low vs. High)	—	—	—	—	—	—	—	0.0006, 11.27 (95% CI 2.64 to 48.12)	0.0002, 11.72 (95% CI 3.01 to 45.67)	0.0006, 9 (95% CI 2.48 to 32.7)
D2-40 stromal expression (Low vs. High)	—	—	—	—	—	—	—	—	<0.0001, infinite (95% CI 16.62 to 7454)	<0.0001, 93 (95% CI 9.96 to 868.16)
CD117-positive mast cell density (Low vs. High)	—	—	—	—	—	—	—	—	—	<0.0001, 279 (95% CI 23.61 to 3298)
MVD (Low vs. High)	—	—	—	—	—	—	—	—	—	—

Abbreviations: CI, confidence interval; MVD, microvessel density; OR, odds ratio. Nodal status is reported as N0 vs. N+. The CD163/CD68 ratio is shown as a surrogate of macrophage polarization at the invasive tumor front. Infinite OR values occurred in comparisons with zero cells in contingency tables and should be interpreted cautiously.

## Data Availability

The data presented in this study are available from the corresponding authors upon reasonable request. The data are not publicly available because they contain information that could compromise patient privacy.

## Supplementary Materials

The following supporting information can be downloaded at: https://www.mdpi.com/article/10.3390/ijms27114971/s1.
