# Supplementary material for: Integrating Tumor Budding and the Invasive-Front Microenvironment in Colorectal Carcinoma: An Exploratory Histopathological Score-Based Association Study
Source: Int J Mol Sci. 2026 May 30;27(11):4971. doi: 10.3390/ijms27114971 (PMC13257210; doi:10.3390/ijms27114971)
Supplement: Supplementary file 1 [file ijms-27-04971-s001.zip › ijms-4309826-supplementary.pdf]

Supplementary Table S1. Benjamini-Hochberg FDR sensitivity analysis

Unadjusted p-values are those reported in Table 2. Values reported in Table 2 as <0.0001 were conservatively entered as 0.0001 for FDR estimation. q < 0.05 was considered retained after FDR correction.

Comparison	Unadjusted p-value	FDR q-value	Retained q < 0.05
Age vs sex	0.78	0.794	No
Age vs grade	0.77	0.794	No
Age vs histological subtype	0.76	0.794	No
Age vs LVI	0.77	0.794	No
Age vs tumor budding	0.28	0.453	No
Age vs nodal status	0.77	0.794	No
Age vs CD163/CD68	0.054	0.102	No
Age vs D2-40	0.55	0.697	No
Age vs CD117	0.78	0.794	No
Age vs MVD	0.57	0.697	No
Sex vs grade	0.37	0.498	No
Sex vs histological subtype	0.35	0.498	No
Sex vs LVI	0.38	0.498	No
Sex vs tumor budding	0.78	0.794	No
Sex vs nodal status	0.38	0.498	No
Sex vs CD163/CD68	0.26	0.433	No
Sex vs D2-40	0.37	0.498	No
Sex vs CD117	0.57	0.697	No
Sex vs MVD	0.78	0.794	No
Grade vs histological subtype	0.32	0.491	No
Grade vs LVI	0.0044	0.010	Yes
Grade vs tumor budding	0.001	0.003	Yes
Grade vs nodal status	0.0044	0.010	Yes
Grade vs CD163/CD68	0.0056	0.012	Yes
Grade vs D2-40	<0.0001	<0.001	Yes
Grade vs CD117	<0.0001	<0.001	Yes
Grade vs MVD	<0.0001	<0.001	Yes
Histological subtype vs LVI	1	1.000	No
Histological subtype vs tumor budding	0.13	0.223	No
Histological subtype vs nodal status	0.33	0.491	No
Histological subtype vs CD163/CD68	0.76	0.794	No
Histological subtype vs D2-40	0.094	0.172	No
Histological subtype vs CD117	0.33	0.491	No
Histological subtype vs MVD	0.12	0.213	No
LVI vs tumor budding	<0.0001	<0.001	Yes
LVI vs nodal status	0.0057	0.012	Yes
LVI vs CD163/CD68	0.038	0.075	No
LVI vs D2-40	0.0044	0.010	Yes
LVI vs CD117	0.0007	0.002	Yes
LVI vs MVD	<0.0001	<0.001	Yes
Tumor budding vs nodal status	0.003	0.008	Yes
Tumor budding vs CD163/CD68	0.0006	0.002	Yes
Tumor budding vs D2-40	<0.0001	<0.001	Yes
Tumor budding vs CD117	<0.0001	<0.001	Yes
Tumor budding vs MVD	<0.0001	<0.001	Yes
Nodal status vs CD163/CD68	0.0005	0.002	Yes
Nodal status vs D2-40	0.0044	0.010	Yes
Nodal status vs CD117	0.0007	0.002	Yes
Nodal status vs MVD	0.006	0.012	Yes
CD163/CD68 vs D2-40	0.0006	0.002	Yes
CD163/CD68 vs CD117	0.0002	<0.001	Yes
CD163/CD68 vs MVD	0.0006	0.002	Yes
D2-40 vs CD117	<0.0001	<0.001	Yes
D2-40 vs MVD	<0.0001	<0.001	Yes
CD117 vs MVD	<0.0001	<0.001	Yes
